# Patient safety education at Japanese medical schools: results of a nationwide survey

**DOI:** 10.1186/1756-0500-5-226

**Published:** 2012-05-10

**Authors:** Shoichi Maeda, Etsuko Kamishiraki, Jay Starkey

**Affiliations:** 1 Graduate School of Health Management, Keio University, 4411 Endo, Fujisawa, Kanagawa, 252-8530, Japan; 2 Graduate School of Social Welfare, University of Kochi, 2751-1 Ike, Kochi City, Kochi, 781-8515, Japan; 3 Dept. of Internal Medicine, University of California, San Diego, 200 W. Arbor Drive # 8425, San Diego, CA, 92103-8425, USA

**Keywords:** Patient safety, Education, Medical school, Nationwide survey, Japan

## Abstract

**Background:**

Patient safety education, including error prevention strategies and management of adverse events, has become a topic of worldwide concern. The importance of the patient safety is also recognized in Japan following two serious medical accidents in 1999. Furthermore, educational curriculum guideline revisions in 2008 by relevant the Ministry of Education includes patient safety as part of the core medical curriculum. However, little is known about the patient safety education in Japanese medical schools partly because a comprehensive study has not yet been conducted in this field. Therefore, we have conducted a nationwide survey in order to clarify the current status of patient safety education at medical schools in Japan.

**Results:**

Response rate was 60.0% (n = 48/80). Ninety-eight-percent of respondents (n = 47/48) reported integration of patient safety education into their curricula. Thirty-nine percent reported devoting less than five hours to the topic. All schools that teach patient safety reported use of lecture based teaching methods while few used alternative methods, such as role-playing or in-hospital training. Topics related to medical error theory and legal ramifications of error are widely taught while practical topics related to error analysis such as root cause analysis are less often covered.

**Conclusions:**

Based on responses to our survey, most Japanese medical schools have incorporated the topic of patient safety into their curricula. However, the number of hours devoted to the patient safety education is far from the sufficient level with forty percent of medical schools that devote five hours or less to it. In addition, most medical schools employ only the lecture based learning, lacking diversity in teaching methods. Although most medical schools cover basic error theory, error analysis is taught at fewer schools. We still need to make improvements to our medical safety curricula. We believe that this study has the implications for the rest of the world as a model of what is possible and a sounding board for what topics might be important.

## Background

Patient safety education, including error prevention strategies and management of adverse events, has become a topic of worldwide concern [1-5], but only a minority of programs have formally incorporated patient safety topics into the medical education curriculum [1,6]. Advocates of patient safety continue to insist the reform of curriculum, with the Association of American Medical Colleges (AAMC) publishing the Medical Schools Objectives Project report advocating patient safety and the United Kingdom House of Commons Health Committee acknowledging educational deficiencies and recommending inclusion in basic medical curricula [7,8]. An especially poignant guideline by the World Health Organization (WHO) states, “Building students’ patient safety knowledge needs to occur throughout medical school. Patient safety skills and behaviours should begin as soon as the students enter a hospital, clinic or health service… Medical students, as future clinicians, will need to know how systems impact on the quality and safety of health care, how poor communication can lead to adverse events and much more. Students need to learn how to manage these challenges” [9].

### Concern about patient safety in Japan

#### Turning point

Japan’s public interest in patient safety was sparked by two serious medical accidents in the late ‘90s. In Jan. 1999, at the Yokohama City University Hospital, a patient mix-up resulted in surgeons performing a cardiac surgery on a pulmonary patient and a pulmonary surgery on a cardiac patient. In this case, all medical staff including the surgeons, anesthesiologists, and nurses failed to properly identify the patients. In a separate incident in Feb. 1999 at the Hiroo General Hospital, a nurse mistakenly injected an antiseptic into a patient, who immediately died. The mistake resulted from improper storage and labelling of medications. The nurses and physicians involved in these cases were criminally prosecuted. Soon after, backed by public demand, patient safety began to gain notoriety as an area in need of drastic reform, including error prevention, adverse event management, a model project for error reform, and legal liability for error.

#### Error prevention

Safety advocates began to emphasize the need of education for physicians on medical error theory, such as human factors contributing to error and theories and models of error. In an effort to quantify error rates and systematically analyze errors for prevention measures, the Japan Council for Quality Health Care (JCQHC), an accreditation agency similar to the Joint Commission in the United States, established the “Project to Collect Medical Near-Miss/Adverse Event Information [10].”

More practical topics related to patient safety also gained attention in Japan. Patient relations became a focus of improvement, specifically soliciting feedback from patients about any safety issues they encountered during their hospitalizations. Practical safety habits, such simple things as reading back and confirming orders, gained popularity, too.

#### Management of adverse events

In the U.S., the Harvard University affiliated hospitals published a consensus paper, “When Things Go Wrong” and more recently the “Sorry Works Coalition” is gaining interest [11,12]. In England, the National Health Service initiated the “Being Open Project” in 2006 [13]. The need for hospitals in Japan to become more transparent and share their errors with other institutions for the sake of learning and improvement has also been emphasized. Responding to adverse events when they do occur is now regarded as an important part of patient safety, for example, in patient communication, disclosure, and apology [14].

#### The model project

The government responded to patient safety by establishing a pilot system for dealing with sentinel events leading to patient death. The Ministry of Health, Labour and Welfare (MHLW) launched a “Model Project” for investigation and analysis of healthcare-associated patient deaths in September 2005 [15]. If the regional office accepts a case, the office assembles a 3-person team including physician in the same subspecialty as that involved in the case, a clinical pathologist, and a forensic pathologist to perform autopsy and determine cause of death. A second team interviews hospital staff, reviews the medical record, and encourages the hospital to conduct an internal investigation. Following investigation, a report is issued detailing the medical course of care and conclusions about how the error could have been prevented [16].

#### Legal liabilities

The number of civil litigation on medical malpractice steadily increased year by year. Physicians were also held criminally liable for error. The Yokohama City University Hospital case and the Hiroo General Hospital case were sentinel cases handled through the Japanese criminal legal system, and a number of subsequent cases of medical error have been handled likewise, with the number of healthcare provider criminal prosecutions for medical error leading to patient death has been on the rise since [17].

### Patient safety education at medical schools in Japan

#### Curriculum guideline in Japan

With so much new activity related to patient safety, the need to educate future physicians about such topics was acknowledged, and in 2008 the Japanese Ministry of Education, Culture, Sports, Science & Technology (MEXT) revised their official medical school curriculum guideline, called the Model Core Curriculum, to include patient safety as part of the core medical curriculum [18]. New guidelines are typically adopted by public medical schools and then private medical schools in Japan, and the effects of these guidelines have yet to be seen.

#### Teaching format

When schools do decide to teach patient safety, teaching format becomes a topic of interest. In Japan, lecture based learning is still the norm. Lectures are the most efficient in terms of a single person being able to deliver information to a large group, and alternative teaching methods perhaps require more preparation, teaching staff, and effort. However, physician competency requires both foundational knowledge and the ability to apply problem solving skills to practical situations, and recently in Japan alternative teaching methods such as small group learning, role playing, hospital based practical experiences, and student to student teaching, such as through assigned research topic presentations, are gaining popularity. Teaching format is important to effective learning, and this applies to patient safety education, too.

### Current safety education teaching at medical schools in Japan

Every other year, the Association of Japanese Medical Colleges (AJMC) publishes a report related to medical education curricula that provides some information about medical safety education [19]. From the report, that lists the title of various courses, and in some cases, the number of hours devoted to each course, based on the title of the course we have positive proof that many schools in Japan have specific courses devoted to patient safety education. Approximately 55% of medical schools list a course that is obviously or conceivably related to patient safety, and where indicated, the range of hours devoted was 6 to over 50 h (from 2005–2009). The school reporting the most curricular hours is Yokohama City University, where the heart-lung accident happened in 1999, indicating it devoted 33, 45, and 52.5 curricular hours to medical safety education in 2005, 2007, and 2009, respectively (over the total 6 year curriculum). However, the informal reports lack detail. Most schools do not list the number of curricular hours; for example, in 2009, only 16/80 schools list hours, producing a median of 16.7 h. In some cases it is unclear how relevant a particular course is to patient safety education (e.g. “Legal Medicine”), and the particulars of courses (content, topics, teaching methods, etc.) are not specified.

### The aim of this research

We previously conducted a study to characterize the state of patient safety within the nursing field [20]. However, the current state of patient safety education at medical schools in Japan is not well characterized. We hypothesize that, as thought leaders and early adopters of governmental guidelines, public schools will likely lead private schools in terms of incorporation into the curriculum, and the hours devoted will be greater at public than private institutions. We therefore aim to describe how many hours, what instructional methods, and what specific topics medical schools employ to teach patient safety and if public and private institutions differ in these regards. This information may aid in the decisions of resource allocation and strategy for improving patient safety education in Japan, providing information to the international community about what is achievable in terms of adopting safety education in medical curricula.

## Methods

This is a cross-sectional research study. We developed a structured, anonymous, self-administered survey about curricular incorporation of patient safety, topics covered, hours devoted, teaching methods, testing and teaching materials, school characteristics, and respondent demographics. As with our previous survey about nursing schools [20], we based the questionnaire on the current WHO guidelines [9], the Japanese model core curriculum guidelines for patient safety education [18] and our previous works regarding to the management of adverse events [14].

The list of Japanese medical schools was obtained from the University hospital Medical Information Network (UMIN) website [21]. The survey was mailed via the Japanese postal system to all 80 public and private medical schools in operation as of April 2010. Surveys were addressed to the dean of each school for distribution to the person in charge of patient safety education. Data collection occurred from April 1st to 15th, 2010.

We used JMP8.0 software for statistical analysis. We compared the data for public schools and private schools using chi-squared analysis, unless the expected frequency for a cell was less than five, in which case we used Fisher's exact test. We used the Mann–Whitney *U* test for analyzing class hours. Significance was set at an alpha less than 0.05 and statistically significant differences between public and private medical schools are denoted by †.

## Results

### Participation (Table 1)

**Table 1:** Responses to a 2010 National Survey of Safety Education at Japanese Medical Schools

	**Public**	Private^*^	**Total**
Number of eligible schools (n)	50	30	80
Average students	112.1	110.7	111.6
Respondents (n)	31	17	48
Participation rate (%)	62.0	56.7	60.0

*We considered the National Defense Medical College a private school.

The overall response rate was 60.0% (n = 48/80 of eligible schools), which was equal for public and private institutions with 62.0% (n = 31/50) and 56.7% (n = 17/30) of eligible public and private institutions participating, respectively.

### Patient safety curricular inclusion

Of the respondents, 97.9% (n = 47/48) indicated that their schools cover the topic of patient safety in some form; 29.8% (n = 14/47) reported requiring courses specifically devoted to patient safety.

### Total hours (Figure 1)

**Figure 1:**
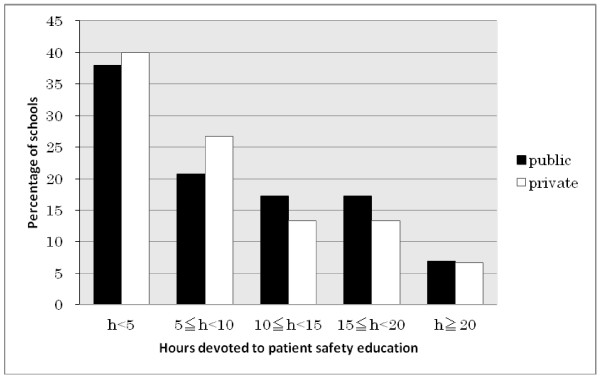
**Hours Devoted to Safety Education.** For statistical analysis, the Mann–Whitney *U* test was used; blank responses were excluded. There were no significant differences between public and private medical schools.

Participants from public and private medical schools reported devoting an average of 9.61 (SD ±9.20) and 8.13 (SD ±6.45) total curricular hours to patient safety education. There were no significant differences between respondents from public and private medical schools in this regard. Of the schools that teach patient safety, 38.6% (n = 17/44, excluding the 3/47 schools that did not indicate number of hours) reported devoting less than five curricular hours to patient safety.

### Teaching methods (Table 2)

**Table 2:** Teaching Methods Utilized for Safety Education

**School Type**	**Public (N = 31)**	**Private (N = 16)**	**Total (N = 47)**
	**n (%)**	**n (%)**	**n (%)**
Lecture	31 (100.0)	16 (100.0)	47 (100.0)
Group discussion	15 (48.4)	4 (25.0)	19 (40.4)
Simulations	5 (16.1)	2 (12.5)	7 (14.9)
Student presentations †	7 (22.6)	0 (0.0)	7 (14.9)
Hospital based learning	5 (16.1)	1 (6.3)	6 (12.8)
Role play	3 (9.7)	2 (12.5)	5 (10.6)
Others	2 (6.5)	1 (6.3)	3 (6.4)

For statistical analysis, the chi-square test or Fisher’s exact test was used; blank responses were excluded.

† *P* < 0.05 comparing public and private medical schools.

All respondents reported using lecture based teaching while few reported adopting other methods such as role-playing or hospital-based hands-on training. Forty percent (n = 19/47) reported using group discussions. There was statistically significant difference in the percentage of respondents who reported using student research presentations as a teaching method at public medical schools (22.6%) versus private medical schools (0%).

### Patient safety education topics by category and topic (Table 3)

**Table 3:** Patient Safety Education Topics by Category and Topic

		**Public (N=31)**	**Private (N=16)**	**Total (N=47)**
		**n (%)**	**n (%)**	**n (%)**
**Hospital safety management**				
	Institutional near-miss/ adverse event reporting	22 (71.0)	11 (68.8)	33 (70.2)
	Committee for patient safety	21 (67.7)	10 (62.5)	31 (66.0)
	Department of patient safety	18 (58.0)	10 (62.5)	28 (59.6)
	Principles of patient safety	18 (58.1)	8 (50.0)	26 (55.3)
	Patient safety officer	20 (64.5)	6 (37.5)	26 (55.3)
	Staff orientation for patient safety	16 (51.6)	6 (37.5)	22 (46.8)
	Investigation committee for adverse events	13 (41.9)	5 (31.3)	18 (38.3)
	Reporting to Japanese Council for Quality Health Care [10]	7 (22.6)	2 (12.5)	9 (19.1)
	Patient relations (patient feedback)	6 (19.4)	3 (18.8)	9 (19.1)
**Medical error theory**				
	Human factors	25 (80.6)	13 (81.3)	38 (80.9)
	Theories and models (Swiss Cheese Model, Heinrich’s Law)	25 (80.6)	11 (68.8)	36 (76.6)
	System factors	21 (67.7)	12 (75.0)	33 (70.2)
	Work environment	19 (61.3)	7 (43.8)	26 (55.3)
**Practical safety**				
	Reporting near-miss/ adverse events	22 (71.0)	12 (75.0)	34 (72.3)
	Verifying patient identity †	24 (77.4)	8 (50.0)	32 (68.1)
	Double-checking	20 (64.5)	7 (43.8)	27 (57.5)
	Communication of near-miss/ adverse events internally	18 (58.1)	7 (43.8)	25 (53.2)
	Identifying risks and developing prevention strategies †	18 (58.1)	5 (31.3)	23 (48.9)
	Standardizing procedures †	19 (61.3)	3 (18.8)	22 (46.8)
	Fail-safe systems	16 (51.6)	5 (31.3)	21 (44.7)
	Object pointing with verbal confirmation †	18 (58.1)	2 (12.5)	20 (42.6)
	Reading back verbal orders †	17 (54.8)	1 (6.3)	18 (38.3)
	Modifying drug names †	14 (45.2)	2 (12.5)	16 (34.2)
	Patient cooperation †	12 (38.7)	2 (12.5)	14 (29.8)
	Concept of fool-proof	11 (35.5)	3 (18.8)	14 (29.8)
	Appropriate documentation of adverse events	11 (35.5)	2 (12.5)	13 (27.7)
	Coherence of documentation of adverse events	10 (32.3)	2 (12.5)	12 (25.5)
	Confirming orders †	9 (29.0)	0 (0.0)	9 (19.2)
**Error analysis**				
	Root Cause Analysis	9 (29.0)	2 (12.5)	11 (23.4)
	Software, Hardware, Environment, and Liveware (SHEL) Model †	7 (25.6)	0 (0.0)	7 (14.9)
	4M-4E	6 (19.4)	0 (0.0)	6 (12.8)
	Failure Mode and Effect Analysis (FMEA)	1 (3.2)	1 (6.3)	2 (4.3)
**Management of adverse events**				
	Patient communication	14 (45.2)	9 (56.3)	23 (48.9)
	Reporting unnatural deaths to the police	13 (41.9)	8 (50.0)	21 (44.7)
	Formulating prevention strategies	15 (48.4)	6 (37.5)	21 (44.7)
	Emergency protocols	14 (45.2)	6 (37.5)	20 (42.6)
	Apology	13 (41.9)	7 (43.8)	20 (42.6)
	Documentation	12 (38.7)	6 (37.5)	18 (38.3)
	Hospital investigation	11 (35.5)	5 (31.3)	16 (34.1)
	Definition of terms	12 (38.7)	4 (25.0)	16 (34.1)
	Transparency/public disclosure	9 (29.0)	5 (31.3)	14 (29.8)
	Preservation of evidence	9 (29.0)	4 (25.0)	13 (27.7)
	Recommending autopsy	8 (25.8)	4 (25.0)	12 (25.5)
	Analyzing medical errors	7 (22.6)	5 (31.3)	12 (25.5)
	Management of medical personnel involved in the adverse event	9 (29.0)	2 (12.5)	11 (23.4)
	Sharing adverse events with other institutions for learning	6 (19.4)	4 (25.0)	10 (21.3)
**Autopsy**				
	Clinical autopsy	18 (58.1)	12 (75.0)	30 (63.8)
	Judicial autopsy	18 (58.1)	10 (62.5)	28 (59.6)
	Administrative autopsy	11 (35.5)	8 (50.0)	19 (40.4)
	Model Project for healthcare-associated patient deaths [15]	8 (25.8)	3 (18.8)	11 (23.4)
**Legal and societal responsibilities**				
	Civil liabilities	26 (83.9)	12 (75.0)	38 (80.9)
	Criminal prosecution	25 (80.6)	12 (75.0)	37 (78.7)
	Societal responsibilities	20 (64.5)	11 (68.8)	31 (66.0)
	Administrative penalties on the individual	19 (61.3)	10 (62.5)	29 (61.7)
	Administrative penalties on the institution/system	11 (35.5)	7 (43.8)	18 (28.3)

Questionnaire topics were selected based on the current WHO guidelines, the Japanese model core curriculum guidelines for patient safety education, and our previous survey regarding the management of adverse events”.

For statistical analysis, the chi-square test or Fisher’s exact test was used; blank responses were excluded.

† *P* < 0.05 comparing public and private medical schools.

More than three quarters of respondents reported covering the topic of human factors, theories and models of error, civil litigation, and criminal prosecution. Less than one quarter of respondents reported covering the JCQHC reporting system [10], patient relations (patient feedback), confirming orders, all topics related to theories of error analysis, management of medical personnel involved in adverse events, sharing adverse events with other institutions for learning, and the Model Project for healthcare-associated patient deaths [15].

## Discussion

### Patient safety curricular inclusion

Almost all of respondents reported that their medical school has incorporated some form of patient safety education into the curricula. The result of this survey is good news for other countries that are also working to introduce the topic of patient safety into formal education since it shows this is an achievable goal. While we assumed that differences would exist between public and private institutions in terms of adoption of medical safety into their curricula, this was not the case. Perhaps the stereotype that public institutions are more affected by government guidelines and are thought leaders is unfounded. Additionally, the societal pressure to address medical safety and the general global milieu around this topic may have even greater influence on medical schools in the adoption of medical safety than actual guidelines.

### Total hours (Figure 1)

Figure 1 shows that few schools devoted more than 20 h to the patient safety education. We believe that at least one educational unit, defined in Japan as 15 periods of 90 min, or 22.5 h of education time, would be required at the minimum to cover all these topics, and the current status is far from the sufficient level. While we acknowledge this is a significant amount time, the significance of medical error is more than enough to justify a large investment.

### Teaching methods (Table 2)

Simulations, student to student research presentations, hospital based hands-on training, and role play have been employed in few medical schools while traditional lecture-based education has been employed in all medical schools that teach patient safety. The best method for teaching patient safety has not been established, but it is unlikely to be lecture format. We believe small group problem based learning would be superior to lecturing. Future research could focus on optimal teaching methods.

### Patient safety topics (Table 3)

#### Topics covered by more than three quarters of schools

More than three quarters of schools covered three topics: 1) human factors, 2) theories and models of error, and 3) civil liabilities and criminal prosecution.

#### Human factors, theories and models of error

The Yokohama and Hiroo cases led investigators emphasize the need of education on medical error theory, such as human factors contributing to error and theories and models of error. This may be why so many medical schools cover the topics of human factors, theories, and models of error.

#### Civil litigation and criminal prosecution

In Japan, public distrust towards clinical medicine was heightened by mass media coverage of the two serious medical accidents in 1999. Since then the number of civil litigations that relate to medical accidents steadily increased. While criminal prosecution occurs in other countries [22,23], in Japan, the number of cases is exceptional [17]. A recent case of medication error led to not only the attending physician, but also the department head being indicted and convicted, receiving sentences of 18 months imprisonment with a 3 year stay of execution and 12 months imprisonment with a 3 year stay of execution, respectively. It is likely the concern for legal ramifications of medical error that so many schools cover these topics.

### Topics covered by less than one quarter of schools

Less than one quarter of schools covered the topic of reporting to JCQHC, patient relations (patient feedback), confirming orders, all topics related to error analysis, management of medical personnel involved in the adverse event, sharing adverse events with other institutions for learning and Model Project for healthcare-associated patient deaths.

#### Reporting to JCQHC, patient relations (patient feedback)

In Japan, since 2002 Health Service Law requires high-level medical facilities, such as university hospitals, to establish the department for patient relation (patient feedback). Since 2004, the same law also requires that high-level medical facilities report medical adverse events to the JCQHC. However, these obligations are not imposed on all medical facilities. In addition, reporting to JCQHC and running a department for patient relations (patient feedback) are considered the responsibility of risk managers, not physicians, and this may be why many medical schools tend to skip this topic.

#### Confirming orders

Generally speaking, in Japan, physicians give medical orders to other medical staff such as nurses and pharmacists, and the medical staff is expected to confirm the orders. Medical orders are usually not addressed to physicians. This may be why many medical schools don’t cover this topic. However, given that physicians also give other physicians orders, it seems that confirmation would be a reasonable skill for physicians to have as well.

#### Error analysis

Topics related to error analysis are advanced and somewhat in-depth topics that require expertise and experience to teach effectively. Lack of medical educators trained in this area may be the reason why many medical schools do not cover this topic.

#### Management of medical personnel involved in the adverse event, sharing adverse events with other institutions for learning

Management of medical personnel involved in the adverse event and sharing adverse events with other institutions for learning are somewhat more within the domain of risk managers, not physicians, and thus many medical schools do not cover these topics.

#### Model project for healthcare-associated patient deaths

The MHLW program has not been widely implemented, and this project is carried out in only 10 areas. This may be why many medical schools do not cover this topic.

### Limitations

This study has a number of limitations. First, although a 60% response rate is relatively high for survey research [24-26], it is likely that non-responders differ significantly from responders, and specifically programs that do not include patient safety education in their curricula may not have responded. We have likely overestimated the true number of programs that have incorporated medical safety into the curricula. On the other hand, our results are on par with the less detailed, biennial reports by the AJMC, and we can conclude that the true number is likely upwards of 60%. Second, we acknowledge that ultimately the effectiveness of medical safety education must be measured in patient safety outcomes and indirectly through test performance rather than curricular hours and content. However, we think that understanding the curricula is an important first step in achieving these goals.

## Conclusions

Based on responses to our cross-sectional survey, most Japanese medical schools have incorporated the topic of patient safety into their curricula. While we assumed that public schools would lead private schools in terms of medical safety, they did not differ significantly in this regard. Many schools, however, devote less than 5 curricular hours to the topic, hardly enough to adequately cover even rudimentary topics, and this was reflected in the reported sparse coverage of more advanced topics. All schools employ lecture-based teaching while fewer use other, likely more effective formats such as role-playing. Room for improvement in patient safety education by increasing hours devoted, diversifying teaching methods, and adding new topics such as error analysis is abundant.

This paper investigates patient safety education in Japan, but we believe that it has implications for the rest of the world as well, both as a model of what is possible, and as a sounding board for what topics might be important for inclusion when discussing patient safety education and how these topics might best be presented. We must not forget that improved patient outcomes through widespread change in day-to-day medical practice is the ultimate goal of patient safety education.

## Competing interests

The authors declare that they have no competing interests.

## Authors’ contributions

SM conceived of the study and designed the study. EK SM participated in the statistical analysis. SM JS also contributed to the manuscript. All authors read and approved the final manuscript.

## Funding

This work was supported by the Japan Medical Association Research Institute.
